# Lupus Erythematosus Tumidus Mimicking Erythema Nodosum: A Diagnostic Challenge

**DOI:** 10.7759/cureus.104616

**Published:** 2026-03-03

**Authors:** Thanda Aung, Kaitlin Eblen, Joshua Pierce, Gregory Gates

**Affiliations:** 1 Rheumatology, University of California, Los Angeles, David Geffen School of Medicine, Los Angeles, USA; 2 Dermatopathology, University of California, Los Angeles, David Geffen School of Medicine, Los Angeles, USA

**Keywords:** erythema nodosum, hydroxychloroquine, lupus erythematosus tumidus, plaquenil, systemic lupus erythematosus, tumid lupus

## Abstract

Lupus erythematosus tumidus (LET) is an uncommon variant of cutaneous lupus that may clinically resemble other inflammatory dermatoses, leading to diagnostic delay. We report the case of a 36-year-old woman with a four-year history of recurrent erythematous nodules initially diagnosed as erythema nodosum. Serologic evaluation revealed a strongly positive antinuclear antibody (1:1280) and elevated anti-double-stranded DNA (1:160) titers. Given persistent symptoms and incongruent clinical findings, a repeat skin biopsy was performed and confirmed the diagnosis of tumid lupus. Treatment with hydroxychloroquine resulted in complete resolution of cutaneous lesions and normalization of serologic markers. This case highlights the potential for atypical presentations of LET and underscores the importance of histopathologic confirmation when the clinical course is inconsistent with the initial diagnosis.

## Introduction

Lupus erythematosus tumidus (LET), recognized in the 2004 Düsseldorf Classification as intermittent cutaneous lupus erythematosus, is a distinct subtype within the spectrum of cutaneous lupus erythematosus (CLE) [1]. CLE comprises acute, subacute, and chronic variants; LET is considered a separate entity because of its characteristic clinical and histopathologic features and minimal epidermal involvement. Unlike discoid lupus erythematosus, LET lesions resolve without scarring or dyspigmentation.

Clinically, LET presents as erythematous, edematous papules or plaques, most commonly affecting sun-exposed areas such as the face, neck, and upper trunk. Photosensitivity is a prominent feature, occurring in approximately 70% of patients [2]. Serologic abnormalities are less frequent than in other CLE subtypes, and progression to systemic lupus erythematosus (SLE) is relatively uncommon. Histopathologically, LET is characterized by superficial and deep perivascular and periadnexal lymphocytic infiltrates with prominent interstitial dermal mucin deposition and minimal to absent dermal-epidermal junction involvement [1]. The absence of significant interface dermatitis helps distinguish LET from other CLE variants. The clinical course is generally benign, although relapses are common, particularly following UV exposure [1]. Management centers on photoprotection and topical therapies, with systemic antimalarials reserved for extensive or refractory disease.

Despite its distinct clinicopathologic profile, LET may present atypically and mimic other inflammatory conditions. We report a case of LET presenting as recurrent erythematous nodules initially misdiagnosed as erythema nodosum, underscoring the importance of clinicopathologic correlation.

## Case presentation

A 36-year-old Asian woman presented to our rheumatology clinic in 2023 with a four-year history of recurrent pruritic erythematous nodules. Notably, symptom onset began in 2019 shortly after relocating to Arizona, a region with high UV exposure. She subsequently developed monthly episodes of raised, pruritic rashes predominantly affecting sun-exposed areas, including the arms and back, with partial response to topical corticosteroids. An initial skin biopsy in 2019 demonstrated mild perivascular lymphocytic inflammation without a definitive diagnosis.

Given her Arizona residence and clinical pattern, erythema nodosum secondary to coccidioidomycosis was suspected. A 2020 biopsy from the right lower extremity demonstrated septal panniculitis with mild perivascular lymphocytic infiltrate in the dermis and septal inflammatory infiltrate containing histiocytes, lymphocytes, multinucleated giant cells, and karyorrhectic debris in the subcutis, along with septal thickening and fibrosis extending into adjacent fat lobules. However, coccidioidomycosis serologies and chest X-ray were negative. She was subsequently lost to follow-up after moving to California.

**Figure 1:**
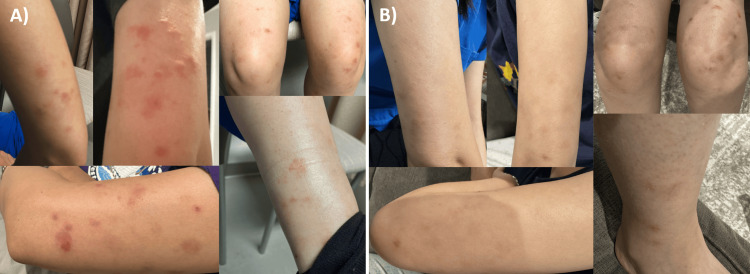
Clinical photographs demonstrating improvement after 2.5 years of hydroxychloroquine (A) July 2023 pretreatment: erythematous, indurated plaques on bilateral arms, thighs, and ankles. (B) January 2026 post-treatment: marked resolution of lesions.

At the initial evaluation in 2023, she denied malar rash, joint pain, alopecia, sicca symptoms, or miscarriage. Examination revealed erythematous and hyperpigmented macules and papules on the upper arms, thighs, and shins (Figure 1A). Laboratory evaluation showed normal CBC and comprehensive metabolic panel results. Autoimmune workup revealed a markedly elevated antinuclear antibody (ANA) titer of 1:1280 (normal <1:80) and a positive anti-double-stranded DNA (anti-dsDNA) titer of 1:160 (normal <1:10). Histone antibody, anti-Sm/RNP, complement levels (complement 3 (C3) and complement 4 (4)), cryocrit, and antineutrophil cytoplasmic antibodies were negative or within normal limits. Extended workup for scleroderma (Scl-70, anti-centromere B), myositis (Jo-1, Mi-2), and sarcoidosis (angiotensin-converting enzyme) was negative. Antiphospholipid antibodies (dilute Russell viper venom time, anticardiolipin IgG/IgM, anti-β2-glycoprotein), inflammatory markers (erythrocyte sedimentation rate and CRP), and QuantiFERON-TB Gold were negative (Table 1). Urinalysis was normal, and she did not meet the 2019 American College of Rheumatology (ACR)/European Alliance of Associations for Rheumatology (EULAR) SLE classification criteria.

**Table 1:** Key laboratory values ACE, angiotensin-converting enzyme; ANA, antinuclear antibodies; ANCA, antineutrophil cytoplasmic antibodies; C3, complement 3; C4, complement 4; DRVVT, dilute Russell viper venom time; dsDNA, double-stranded DNA; ESR, erythrocyte sedimentation rate; H, high; MTB, Mycobacterium tuberculosis; Sm/RNP, Smith/ribonucleoprotein

Pertinent lab data	Patient’s lab values	Reference range
ESR (mm/hr)	15	≤25
CRP (mg/dL)	<0.3	<0.8
ANA Ab titer	≥1:1280 (H)	<1:80
Rheumatoid factor (IU/mL)	<10	<25
Cyclic citrullinated peptide Ab IgG (units)	3	<19
dsDNA Ab EIA (IU/mL)	≤200	≤200
nDNA (*Crithidia*) Ab IFA (titer)	1:160 (H)	<1:10
Sm/RNP Ab (U)	<20	<20
Histone IgG Ab (AI)	<1.0	<1.0
C3 (mg/dL)	106	86-175
C4 (mg/dL)	24	10-40
IgG1 (mg/dL)	795	240-1118
IgG2 (mg/dL)	386	124-549
IgG3 (mg/dL)	146 (H)	21-134
IgG4 (mg/dL)	53	1-123
Cardiolipin IgA (CU)	<20.0	≤20
Cardiolipin IgG (CU)	<20.0	≤20
Cardiolipin IgM (CU)	<20.0	≤20
β2-glycoprotein Ab (SGU)	<10	≤20
DRVVT	Negative	Negative
Cryocrit	Negative	Negative
Scl-70 (AU/mL)	2	<29
Centromere B Ab (AI)	<1.0	<1.0
Jo-1 (AU/mL)	2	<29
Mi-2	Negative	Negative
C-ANCA (titer)	<1:20	<1:20
P-ANCA (titer)	<1:20	<1:20
ACE (U/L)	25	9-67
MTB-QuantiFERON-Gold ELISA	Negative	Negative

**Figure 2:**
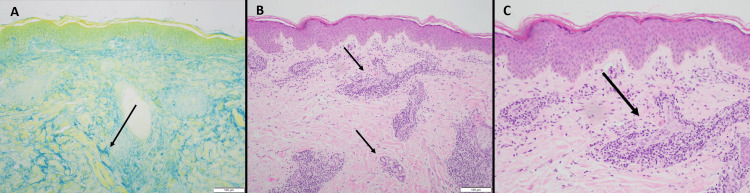
Histopathology of LET (A) Alcian blue stain (100×): increased interstitial dermal mucin, characteristic of tumid lupus (black arrow). (B) H&E stain (100×): uninvolved epidermis with superficial and deep perivascular/periadnexal lymphocyte-predominant inflammation with rare plasma cells and eosinophils (black arrows). (C) Close-up of panel B. LET, lupus erythematosus tumidus

A third biopsy in July 2023 from the right arm showed an intact epidermis with superficial and deep perivascular and periadnexal lymphocytic infiltrates, increased dermal mucin, and mild interface changes, findings consistent with tumid lupus (Figure 2).

Repeat testing during a skin flare showed an increased anti-dsDNA titer (1:160 to 1:640). We initiated hydroxychloroquine 200 mg daily (body weight 50 kg) for cutaneous lupus with concerning serologic features. Within eight weeks, she noted reduced frequency and severity of rashes, with continued improvement through January 2026 (Figure 1B). Her IgG3 level normalized (146-127 mg/dL after 12 weeks). She continues hydroxychloroquine with occasional mild breakthrough flares managed with topical steroids. Her dsDNA titer improved from a peak of 1:640 to 1:80 by December 2025. She has no evidence of systemic lupus, and C3 and C4 remain normal.

## Discussion

This case highlights the diagnostic complexity of LET, particularly in the presence of serologic abnormalities suggestive of SLE. Notably, the patient exhibited no evidence of systemic involvement, with manifestations limited to cutaneous findings and positive serologies. The diagnosis was established based on the characteristic clinical presentation and distinctive histopathologic features. The initial diagnosis of erythema nodosum was reasonable given endemic coccidioidomycosis exposure and the nodular presentation. Garcia et al. recently described a similar case in which presumed erythema nodosum was ultimately identified as LET on biopsy [3]. LET also shares histopathologic features with Jessner lymphocytic infiltrate, polymorphous light eruption, and reticular erythematous mucinosis, contributing to diagnostic confusion [1]. LET rarely coexists with SLE. Stead et al. reported two such cases, and Hajji et al. described biopsy-proven LET in a patient meeting the 2019 EULAR/ACR SLE criteria with class V lupus nephritis [4,5].

Serologic profiles help distinguish LET from SLE. LET typically shows low ANA positivity and rarely demonstrates positive anti-dsDNA or anti-Sm/RNP antibodies. Verdelli et al.’s 2018 study of 108 LET patients found 43.5% were ANA-positive, 22.2% anti-SSA/Ro-positive, and only 3.7% anti-dsDNA or anti-Sm/RNP-positive [6]. Patsinakidis et al. reported among 100 LET patients that 25% were ANA-positive, 6% anti-SSA/Ro-positive, and 1% anti-Sm/RNP-positive [7]. Our patient’s ANA titer of 1:1280 is notably high, raising concern for SLE, although her clinical presentation remained limited to the skin. A high-titer ANA warrants a comprehensive workup, including lupus serologies, CBC, urinalysis, inflammatory markers, and complement levels.

Hydroxychloroquine is central to SLE management and may reduce progression from cutaneous to systemic disease. Bar et al.’s 2025 study of 286 patients with isolated cutaneous lupus found an 87% risk reduction in SLE development over time (hazard ratio: 0.13; 95% CI, 0.06-0.27; P < 0.001) with hydroxychloroquine compared with topical therapies alone [8]. Kreuter et al.’s study of 36 multifocal LET patients showed that 61% achieved complete or near-complete clearance with antimalarial therapy [9]. Importantly, these patients lacked positive ANA or SLE criteria, suggesting benefit even in skin-limited disease. Our patient’s improvement on hydroxychloroquine confirms the diagnosis of LET and demonstrates the therapy’s efficacy.

## Conclusions

LET can present with atypical features that mimic erythema nodosum and other dermatoses. This patient’s excellent response to hydroxychloroquine supports the use of antimalarial therapy in LET, even in the presence of elevated serologic markers. Cases with high-titer ANA and positive lupus serologies warrant ongoing vigilance for systemic involvement, despite initially skin-limited disease. A broad differential diagnosis, histopathologic confirmation, and thorough serologic evaluation are essential when initial diagnoses fail to account for persistent cutaneous findings.
